# Contraceptive Utilization and Associated Factors among HIV Positive Women on Chronic Follow Up Care in Tigray Region, Northern Ethiopia: A Cross Sectional Study

**DOI:** 10.1371/journal.pone.0094682

**Published:** 2014-04-17

**Authors:** Yohannes Adama Melaku, Ejigu Gebeye Zeleke

**Affiliations:** 1 Mekelle University, College of Health Sciences, Department of Public Health, Mekelle, Ethiopia; 2 University of Gondar, Institute of Public Health, Department of Epidemiology and Biostatistics, Gondar, Ethiopia; Catalan Health Institute, Spain

## Abstract

**Background:**

In sub-Sahara Africa, more than 60% of all new HIV infections are occurring in women, infants and young children. Maternal to child transmission is responsible for 90% of childhood HIV infection. Preventing unwanted pregnancy among HIV positive women is imperative to reduce maternal and infant morbidity and mortality.

**Methods:**

A cross-sectional survey was conducted among 964 HIV positive women in selected 12 health centers of Tigray region. In this paper, analysis was restricted only for 847 women who were sexually active and non-pregnant. In each health center the number of study participants was allocated proportionally to the load of HIV positive women in chronic care clinics. The data were entered into EpiData version 3.1, and cleaned and analyzed using Stata version 11.1. Descriptive summary of data and logistic regression were used to identify possible predictors using odds ratio with 95% confidence interval and P-value of 0.05.

**Findings:**

Three hundred ninety four (46.5%) of all HIV positive women had intension to have more children. Three hundred seventy five (44.3%) were using contraceptive methods at time of survey. Injectable (70.7%) and male condom (47.6%) were most commonly used type of contraceptives. In the multivariable analysis, women who were urban dwellers (AOR = 2.55; 95% CI: 1.27, 5.02), completed primary education (AOR = 2.27; 95% CI: 1.12, 2.86) and those openly discussed about contraceptive methods with their husbands or sexual partners (AOR = 6.3; 95% CI: 3.42, 11.76) were more likely to use contraceptive. Women who have one or more living children were also more likely to use contraceptive compared with women with no child.

**Conclusion:**

Less than half of women used contraceptive methods. The use of condoms could impact unintended pregnancies and reduced risks of vertical and sexual transmission. Efforts to increase contraceptive utilization focusing on the barrier methods should be strengthen in HIV/AIDS chronic care units.

## Background

It is estimated that 33 million people are currently living with HIV/AIDS worldwide. Sub-Sahara Africa is home of 60% of people living with HIV/AIDS and more than half of these population groups are females [1]. Studies estimated that 75% of HIV positive people are of reproductive age worldwide [2]. According to 2011 Ethiopian Demographic and Health Survey (EDHS) estimate, the prevalence of HIV in Ethiopia was 1.5% among the population group of age 15–49 years and he figure among women was significantly higher (1.9%) than men (1.0%) [3].

Importantly, HIV infection is linked with women's sexual and reproductive health, because it is not only transmitted by sexual contact but also more than half of the 2.6 million new infections globally were among women [4]. As a result, there is growing recognition of the reproductive decision faced by HIV infected individuals. Studies in both developed and developing countries have suggested that many HIV positive women continue to desire children despite the knowledge of their HIV status [5], [6], [7], [8]. Hence, meeting women's sexual and reproductive health (SRH) needs ensures women have control over their reproductive lives, as well as contributes to public health by reducing maternal and infant mortality and morbidity [9].

Unintended pregnancy is a common problem in both HIV-positive and HIV negative women. A study conducted in Swaziland has indicated that 69.2% of women reported that their recent pregnancy was unintended with no difference in sero-status. However, the rate of unwanted pregnancy was found to be significantly higher in HIV-positive women than their counterparts (20.7% versus 13.5%) [10]. Previous similar studies indicated that the rate of unintended pregnancy is also high among HIV-positive women [11], [12]. Enabling women living with HIV to avoid unintended pregnancy can reduce vertical transmission of HIV and maternal mortality associated with HIV infection [13]. As a result, a component of the World Health Organization (WHO) comprehensive strategy 2011–2015 for Prevention of Mother-to-Child Transmission (PMTCT) is to increase contraceptive use among HIV positive women who wish to use it [14]. However, the prevalence of contraceptive utilization level varies in different settings with low ceiling [10], [11], [15]–[20].

Studies have reported that most women have preferred condom as method of pregnancy prevention. In studies, the most common type of contraceptive used was condom among 7% to 90% of women [15]–[21]. On the other hand, a study in South Africa reported that most women were using short acting method, primarily injectable (70.2%) [11]. Other studies demonstrated that utilization of injectable (4.1%) and pills (3.3%) was found to be low in rural Uganda [17] for different perceived reasons by women [21]. HIV positive women have different reasons and factors influencing their contraceptive utilization unlike their negative counterparts. Open discussion about family planning with health workers and spouse [18], disclosure of HIV sero-status and discussion on fertility issue [19], being married and older age of women [22], were found to be positively associated with current family planning utilization. In regard to the association between education and contraceptive utilization among HIV-positive women, contradicting findings were reported in Uganda and Kenya [18], [23].

Enabling women living with HIV to avoid unintended pregnancy and use of contraceptive methods reduces vertical transmission of HIV and further morbidity and mortality of mothers and children [9]. In particular, contraceptive use averts 19.7% of infections and 13.1% of deaths [24].

In settings where HIV prevalence is high, management of sexual and reproductive health of HIV-infected women is critical to reduce HIV transmission and maternal mortality. However, family planning utilization and factors associated with it have not been well understood in resource limiting settings like Ethiopia.

Therefore, the main aim of this study was to describe family planning utilization which will help in estimating the family planning needs of HIV positive women and which in turn could help to prepare the necessary resources and flourish programs for better reproductive health services. The other main purpose of the current study was addressing the knowledge gap with regard to factors associated with family planning utilization among HIV positive women. Understanding the factors benefit in a way that patients as well as care givers intervene on those factors. This study is also believed to benefit many concerned stakeholders in decision making and policy development.

## Methods

### Study area and period

Tigray region has an estimated total population of 4,664,071, of which 2,367,032 are females. More than 80% of the population is estimated to be rural inhabitants [25]. According the regional health bureau report of 2012, Tigray had 14 hospitals, 205 health centers and 552 health posts [26].

Antenatal care surveillance data in 2009 reported that the HIV prevalence in Tigray was 2.2% (5.0% urban and 1.3% rural) [27]. The 2011 EDHS estimated that overall HIV adult prevalence (15–49 years) to be 1.8% (1.9% among women and 1.0% among men) [3]. In 2012, the Federal HIV/AIDS Prevention and Control Office (HAPCO) estimated that there were about 56,900 HIV positive individuals in the region [28]. HIV prevalence in Tigray varies widely across zones from 0.4% (Central zone) to 2.2% (Western) [29].

In the region there were 60 health centers which were supported by Ethiopian Network of HIV/AIDS Treatment, Care and Support (ENHAT-CS). All health centers provide services including HIV prevention, testing, treatment and care and all family planning methods free-of-charge. Family planning services include barrier, oral, and injectable contraceptive methods as well as family planning counseling from a trained family planning nurse.

**Figure 1:**
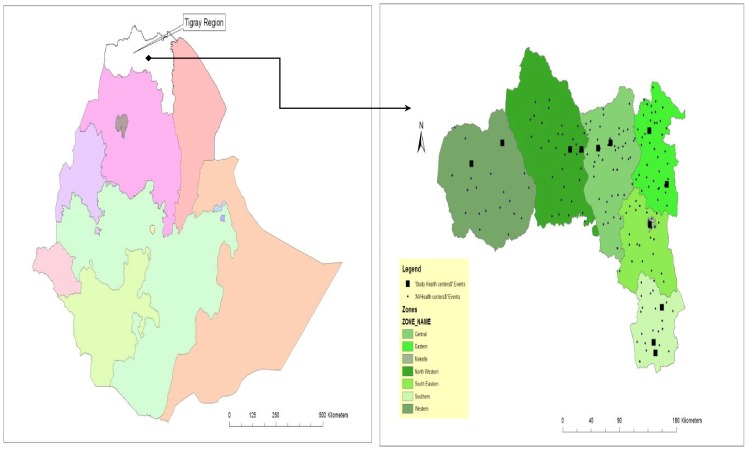
Map of study area (Ethiopia, Tigray region) and location of study health centers.

This study was conducted at chronic follow up care (both pre-ART and ART) clinics in 12 ENHAT-CS supported health centers in Tigray region (**Figure 1**) from May to June 2013.

### Study design

This analysis is based on a cross-sectional survey data of HIV positive women seeking services at ENHAT-CS supported health centers in the region. Medical chart was reviewed to confirm HIV status, ART use history and other medical characteristics of HIV positive women.

### Eligibility criteria

To be eligible to participate in the overall study, women were required to be 15–49 years of age, attending a chronic follow up care, competent to give informed consent, and willing to allow medical record review for the purposes of confirming HIV sero-status and other medical histories. We considered a woman to be ART user if she was taking ART at the time of interview. We considered woman to be pre-ART (not using ART) if she had never taken ART before.

### Sample selection

**Figure 2:**
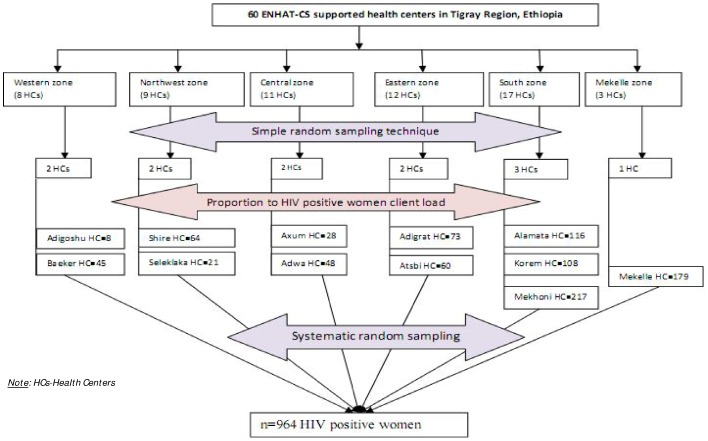
Schematic presentation of sampling procedure.

Twenty percent of ENHAT-CS supported health centers were selected with simple random sampling technique using lottery method. The numbers of health centers in each administrative zone (6 zones of the region) were determined proportionally considering the total number of ENHAT-CS supported health centers in each zone. The numbers of study participants in each health center were determined using proportion to population size (HIV positive women registered in the health centers). In this case, the number of women who were HIV positive and were on chronic follow up care was taken in to account to determine the number of samples in each health center (**Figure 2**).

To select each woman in each health center pre-arrival systematic random sampling technique was used. The first woman to contact was selected by lottery method after determining the interval between two study units.

The analysis was restricted to 847 women aged 15–49 years who were sexually active, and were not pregnant. This was done to enhance the comparability of findings with other studies investigating reproductive and sexual health among HIV positive women.

### Data collection procedures and enumerators

After confirming eligibility and seeking written informed consent, participants were asked to complete a 25–35 minute interviewer-administered questionnaire in local language called “Tigrenga”. Approximately 6 women were interviewed daily by a data collector at each heath center from May to June 2013. Data collectors were trained women from the local community who had previous research experience and were holders of Bachelor degree. In each health center, nurses with HIV/AIDS related training reviewed medical records of participants.

### Data collection instruments

The questionnaire assessed socio-demographic characteristics, fertility intentions, fertility history, contraceptive practice, and sexual history. The survey instrument was developed by organizing variables from previously conducted researches [10], [30], [31]. Medical records of HIV positive women were reviewed to confirm HIV status and other medical history, and to obtain all necessary clinical data including CD4 cell counts and WHO stage of disease using data extraction tool (checklist).

### Measures and operational definitions

The primary outcome was self-reported contraceptive use at the time of the survey. *Contraceptive utilization* refers to use of any form of either modern or traditional contraceptive methods to avoid or delay pregnancy. *Current use of contraceptive method* referred to respondents who responded positively for use of contraceptive methods at time of the survey to delay or avoid pregnancy. Contraceptive method queries included male and female condoms (restricted to those reporting “Always” use), injections (depomedroxyprogesterone acetate (DMPA) or norethisterone enantate), oral contraceptive pills, diaphragm, intrauterine devices (IUD), female tubal ligation, hysterectomy, and male partner sterilization. In addition, traditional methods (coitus interrupts and calendar method) were incorporated as part of the responses in the questionnaire. In assessing the contraceptive method profile, dual protection was defined as use of both a barrier contraceptive method (male condom) and use of a hormonal or permanent contraceptive method [32].

The explanatory variables were current receipt of ART, age, education, employment, current sexual partnership, number of living children, fertility intentions, and HIV clinical variables. “Sexual intercourse” were defined to refer specifically to “vaginal-penile penetrative sex between a man and a woman”. We define *HIV-positive women on chronic care* as women who had at least one visit to the selected pre-ART or ART units for care who may be receiving ART or not. *Fertility intention* was defined as HIV positive women on chronic follow up care would like to have child/children in the future.

### Data quality control, management and analysis

First code was given to the completed questionnaires and then data were entered into Epidata Version 3.1 database and transferred to STATA version 11.1 (Stata Corporation, College Station, TX, USA) statistical packages for analysis.

Pretest of questionnaire was conducted out of study health centers. Supervisors had checked filled questionnaires and checklists for consistency, completeness and accuracy on daily base for all clients. Supportive supervision by investigators was also rendered for each data collectors and supervisors.

Cleaning of data was done to check the consistency and completeness of the data set. Frequencies, proportions and summary statistics were used to describe the study population in relation to relevant variables. Both bivariable and multivariable logistic regression were used to identify significant predictors. The degree of association between independent and dependent variables was assessed using odds ratio with 95% confidence interval. Differences in contraceptive use between groups were reported using Pearson's chi-square or score test for trends of odds for categorical variables. After testing for co-linearity [33] and interaction [34], all covariates with statistically significant associations in the bivariable analysis were included in multivariable logistic regression models to obtain adjusted estimates of the association between covariates and contraceptive use. All statistical tests were two-sided and considered statistical significant at P value of 0.05. ArcGIS version 10.0 was used to map study area and locate health centers.

### Ethics statement

Information letters and written consent forms were available in English and “Tigrenga”. Ethical clearance was obtained from the Institutional Review Board of Mekelle University, College of Health Sciences. The members of Institutional Review Board were nominated by the management body of Mekelle University and consist of experts in the field of subject matter, Medical ethics and statistics. Permission letters were gained from Tigray regional health bureau, respective district health offices and health centers.

After explaining the objective and contents of the study, written consent has been obtained from each respondent or next-of-kin when the respondent were under 18 years of age. Some of the study participants were unable to read and write. In this case, trained interviewers fully explained the purpose, process, benefits, and risks to all study participants before consent was obtained and inked index finger print was used as a signature. This consent procedure was reviewed and approved by Mekelle University College of Health Sciences Institutional Review Board. The information collected was purely used for research works and name of respondents has remained anonymous.

All the interviews with study participants were made with strict privacy and assuring confidentiality. Each interview was conducted in a separate room which was prepared for this purpose. The participants were told that they have the right to either refuse to participate in the study or withdraw after responding for some of the questions. Respondents' name and identity were not recorded, included and linked in the questionnaire. Data were kept confidential by locking in boxes with key and by password in the computer to avoid access of the data by third party. Only card numbers of the clients were used to review secondary data and link with the primary data of the clients. Similarly, the names of the clients have not been reviewed and were not recorded. The collected data also were not linked with the individual identity at all level of data processing and analysis.

## Results

Of 964 women approached for participation, all consented, interviewed and underwent medical record review (participation rate = 100%). Analysis was done for 847 sexually active, non-pregnant and age 15–49 years of old women.

### Socio-demographic characteristics

Majority, 242 (28.6%) of the study participants were in the age between 30 to 34 years and median age was 30 (Inter quartile range (IQR) = 27, 35). Three-quarters (75.6%) of the participants were from urban areas. Of all study participants 92.9% and 91.4% were found in the ethnic group of “Tigrie” and were followers of orthodox Christianity, respectively. About three-fifths (58.4%) of women were illiterate. Housewives, daily laborers and farmers, each accounted for more than three-fifths (21.3%, 21.6% and 19.4%, respectively) of all occupations of the study participants. More than two-fifths of the participants were married at the time of survey. Functional television and radio were present in less than a third (32.4%) and less than half (44.6%) of the study participants' house, respectively (**Table 1**).

**Table 1:** Socio demographic characteristics of HIV positive women of reproductive age in Tigray region, northern Ethiopia, 2013.

Characteristics	Categories	Frequency(n)	Percent (%)
Health centers providing care	Alamata	105	12.4
	Mehoni	177	20.9
	Korem	102	12.0
	Mekelle	168	19.8
	Atsbi	49	5.8
	Adigrat	65	7.7
	Adwa	44	5.2
	Axum	23	2.7
	Selklaka	18	2.1
	Shire	53	6.3
	Adigoshu	6	0.7
	Beaker	37	4.4
Age at interview in years	15–19	23	2.7
	20–24	84	9.9
	25–29	212	25.0
	30–34	242	28.6
	35–39	176	20.8
	40–44	77	9.1
	45–49	33	3.9
Median = 30, IQR = (27, 35)			
Residence	Urban	640	75.6
	Rural	207	24.4
Ethnicity	Tigray	787	92.9
	Amhara	53	6.3
	Other	7	0.8
Religion	Orthodox	774	91.4
	Muslim	62	7.3
	Other	11	1.3
Level of education	No formal education	495	58.4
	Primary education	226	26.7
	Secondary and above education	126	14.9
Occupation	Unemployed	95	11.2
	Housewife	180	21.3
	Daily laborer	183	21.6
	Farmer	164	19.4
	Merchant	119	14.1
	Employed	70	8.3
	Other	36	4.3
Current marital status	Currently married to single husband	351	41.4
	Currently Partnered (with single sexual partner)	181	21.4
	Have more than one sexual partners	315	37.2
Presence of functional Television in the household	Yes	274	32.4
	No	573	67.7
Presence of functional radio in the household	Yes	378	44.6
	No	469	55.4

### Medical and reproductive health related profile of the participants

The proportion of women who had disclosed their sero-status (45.7%) was significantly higher than those who didn't (36.0%) (P = 0.04). Majority of the respondents (786, 92.8%), reported that their health status was improving. More than half (52.5%) of women had one or two children. Median time since HIV diagnosis and duration of ART use were 3.4 years (IQR = 1.9, 4.6]) and 2.2 years (IQR = 0.4, 3.8), respectively. Almost three-fifths of women were in WHO stage-I and 85.2% of them had disclosed their HIV status for someone. More than half (52.8%) had latest CD4 count > = 350 cells/mm^3^ (Median CD4 count 351 cells/mm^3^, IQR = 234, 520) (**Table 2**).

**Table 2:** Medical and reproductive profile of HIV positive women of reproductive age in Tigray region, northern Ethiopia, 2013.

				Contraceptive use		
Characteristics	Categories	Frequency(n)	%	Yes (%)	No (%)	?^2^ P-value
HIV status disclosed for anyone	Yes	722	85.2	330(45.7)	392(54.3)	0.044@
	No	125	14.8	45(36.0)	80(64.0)	
Perceived current health status	Deteriorating	13	1.5	4(30.8)	9(69.2)	0.6965$
	Same	48	5.7	18(37.5)	30(62.5)	
	Improving	786	92.8	353(44.9)	433(55.1)	
Time since HIV diagnosis (years)	<1	96	11.3	35(36.5)	61(63.5)	0.3649$
	[1–2]	128	15.1	60(46.9)	68(53.1)	
	(2–3]	144	17.0	64(44.4)	80(55.6)	
	(3–4]	169	20.0	76(45.0)	93(55.0)	
	(4–14)	310	36.6	140(45.2)	170(44.8)	
Median year 3.3(IQR = 1.9, 4.6) (min 0.0055, max 13.05)						
Current WHO clinical stage	I	495	58.4	250(50.5)	245(49.5)	<0.001$
	II	147	17.4	61(41.5)	86(58.5)	
	III/IV	205	24.2	64(31.2)	141(68.8)	
Last CD4 count	<200	144	17.0	55(38.2)	89(61.8)	0.7244$
	> = 200–350	256	30.2	127(49.6)	129(50.4)	
	> = 350	447	52.8	193(43.2)	254(46.8)	
Median CD4 count 351 (IQR = 234, 520) (min 0, max 1986)						
On ART	Yes	720	85.0	321(44.6)	399(55.4)	0.666@
	No	127	15.0	54(42.5)	73(57.5)	
Duration of ART use (years)	0 (not started)	127	15.0	54(42.5)	73(57.5)	0.4682$
	<1	153	18.1	74(48.4)	79(51.6)	
	[2–3)	125	14.8	58(46.4)	67(53.6)	
	[3–4)	116	13.7	52(44.8)	64(55.2)	
	[4–10)	326	38.5	137(42.0)	189(48.0)	
Median year 2.2 (IQR = 0.4, 3.8) (min 0, max 9.28)						
On other medication/prophylaxis	Yes	679	80.2	296(43.6)	383(55.4)	0.423@
	No	168	19.8	79(47.0)	89(53.0)	
Total # of living children (n = 829)	None	147	17.4	43(29.3)	104(70.7)	0.0047$
	1–2 children	445	52.5	211(47.4)	234(52.6)	
	3–4 children	207	24.4	98(47.3)	109(52.7)	
	5–7 children	48	5.7	23(47.9)	25(52.1)	
Median # of living children = 2 (IQR = 1,3) (min 0, max 7)						
Fertility intentions	Yes	394	46.5	193(49.0)	201(51.0)	0.010@
	No	453	53.5	182(40.2)	271(59.8)	

# *-number,*

@ *-Pearsonχ2 ,*

$ *-Trend of odds χ^2^,*

*min-minimum, max-maximum.*

One fifth (80.2%) of the participants were on non-ART medication/prophylaxis. More than half (52.5%) of women had one or two children and less than half (46.5%) reported intent to have more children in the future. Contraceptive utilization significantly varied with total number of living children women had (P = 0.0047) and fertility intention (P = 0.010) (**Table 2**).

### Prevalence of contraceptive use and types of contraceptive methods

**Figure 3:**
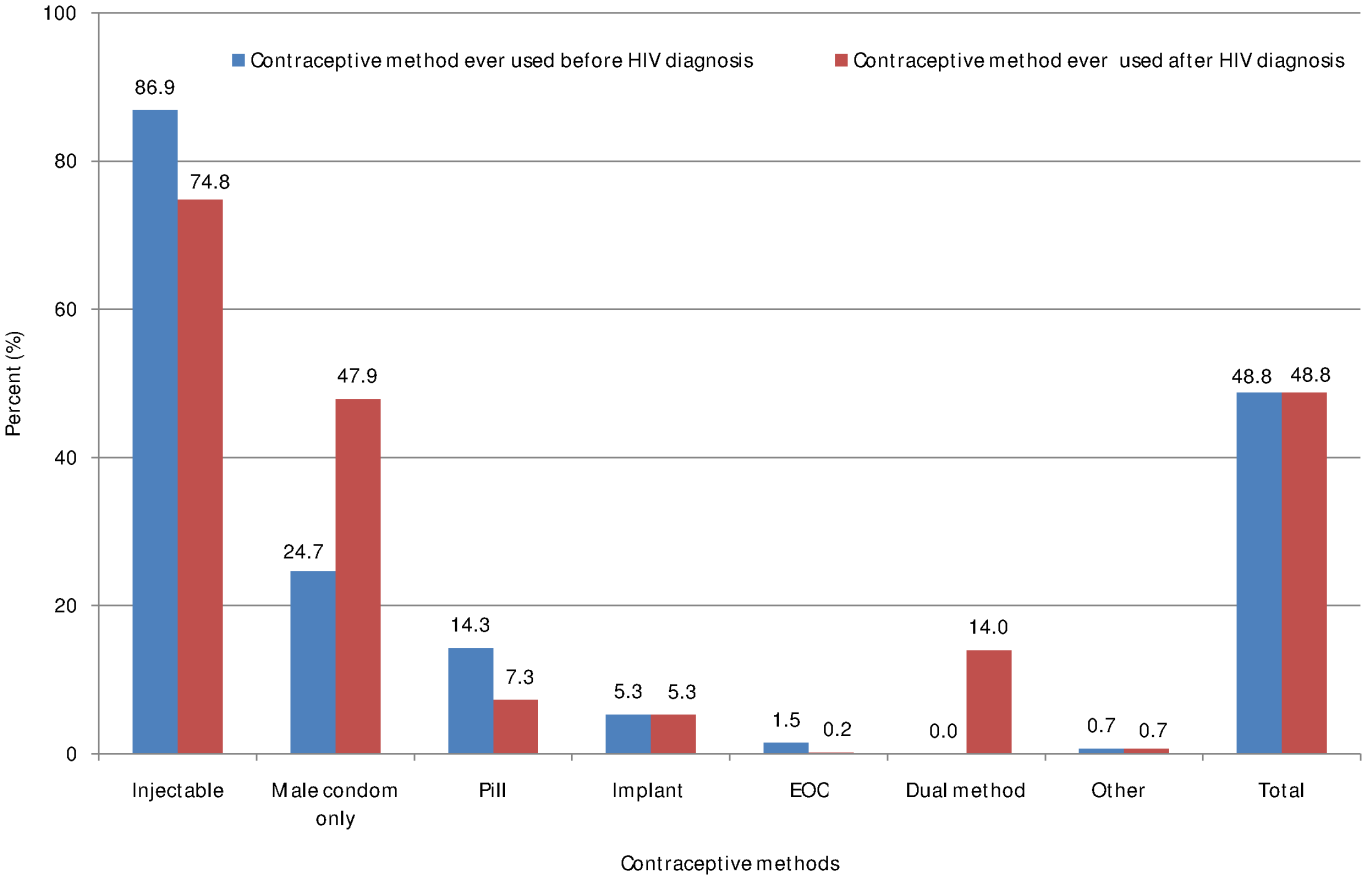
Contraceptive methods ever used before and after HIV diagnosis among HIV positive women of reproductive age in Tigray region, northern Ethiopia, 2013.

**Figure 4:**
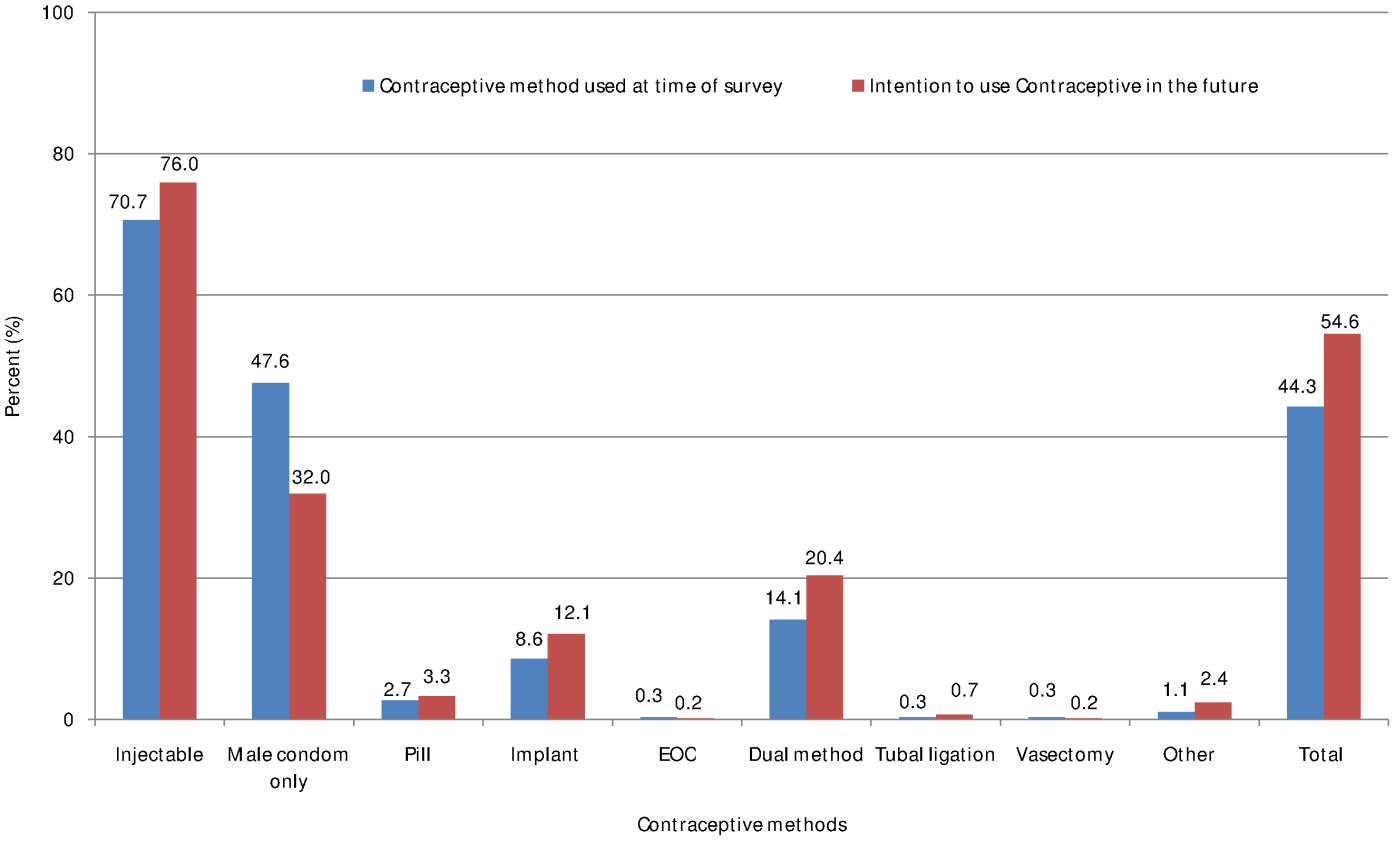
Contraceptive methods used during survey time and future intention among HIV positive women of reproductive age in Tigray region, northern Ethiopia, 2013.

Almost half of the respondents ever used contraceptive methods before and after HIV diagnosis. Exactly similar proportion (413, 48.8%) of women had ever used contraceptive methods before and after HIV diagnosis (**Figure 3**). At time of survey, overall contraceptive prevalence was 44.3%. Four hundred sixty two (54.6%) women had intention to use contraceptive methods in the future (**Figure 4**).

Contraceptive method preferences are shown in **figure 3** **and** **4**. As shown, the most common type of contraceptive ever used before and after HIV diagnosis was injectable (86.9% and 74.8%, respectively). Utilization of male condom has doubled after HIV diagnosis (before diagnosis = 24.7% and after diagnosis = 47.9%) while oral contraceptive pill has decreased almost by half after HIV diagnosis (before diagnosis = 14.3% and after diagnosis = 7.3%). Fourteen percent of women had ever used dual method after HIV diagnosis (**Figure 3**). At time of survey 86.9% and almost half (47.6%) of women were using injectable and male condom, respectively. About 14% of women were using dual method at the time of survey (**Figure 4**).

Generally, utilization of permanent methods (tubal ligation and vasectomy) was very low both before and after HIV diagnosis. However, hormonal and barrier methods were used commonly among HIV positive women (**Figure 3** **and** **4**).

### Determinants of contraceptive use

The unadjusted odds of reporting contraceptive use among ART users and non-users were not significantly different. Age, Educational level, occupation, presence of functional radio and television, number of living children, fertility intention, WHO clinical stage, latest CD4 count, disclosure of status, counseling status and open discussion with sexual partner/husband had crude significant association with contraceptive utilization (**Table 3**).

**Table 3:** Predictors of family planning utilization among HIV positive women of reproductive age in Tigray region, northern Ethiopia, 2013.

		Contraceptive use				
Characteristics	Categories	Yes	No	COR(95% CI)	AOR(95% CI)	P-value
Age (per increase in year)	Median ± SD	30±5.68	31±7.10	0.94(0.92,0.96)**	0.95(0.90,1.00)	0.049
Residence	Urban	321	319	2.85 (2.02, 4.03)**	2.52(1.27,5.02)*	0.008
	Rural	54	153	1.00	1.00	
Educational Level	No formal education	192	303	1.00	1.00	
	Primary education	121	105	1.82(1.32,2.50)**	2.27 (1.12,2.86)*	0.023
	Secondary and above education	62	64	1.53(1.03,2.27)*	1.01(0.45,2.25)	0.981
Occupation	Unemployed	231	231	1.73(1.31,2.28)**	1.69(1.00,2.86)	0.051
	Employed	245	144	1.00	1.00	
Presence of functional Radio	Yes	204	174	2.04(1.55,2.69)*	1.52(0.88,2.61)	0.129
	No	171	298	1.00	1.00	
Presence of functional TV	Yes	161	113	2.39(1.78,3.21)*	1.63(0.85,3.15)	0.142
	No	214	359	1.00	1.00	
Total number of living children	None	43	104	1.00	1.00	
	1–2 children	211	234	2.18(1.46,3.26)**	2.46(1.19,5.11)*	0.015
	3–4 children	98	109	2.18(1.39,3.40)*	4.06(1.57,10.48)*	0.004
	5–7 children	23	25	2.23(1.14,4.34)*	3.95(1.05,14.90)*	0.043
Fertility intention	Yes	193	201	1.43(1.09,1.88)*	0.66(0.35,1.22)	0.185
	No	182	271	1.00	1.00	
WHO clinical stage	I	250	250	2.25(1.59, 3.17)**	1.17(0.60,2.31)	0.644
	II	86	61	1.56(1.00, 2.43)*	1.04(0.45.2.40)	0.922
	III or IV	141	64	1.00	1.00	1.00
Latest CD4 count	<200	55	89	1.00	1.00	
	> = 200 & <350	127	129	1.59(1.05,2.41)*	1.55(0.70,3.43)	0.278
	> = 350	447	254	1.23(0.84,1.81)	0.96(0.46,2.00)	0.912
Disclosed HIV status to anybody	Yes	330	392	1.50(1.01,2.22)*	0.48(0.22,1.06)	0.07
	No	45	80	1.00	1.00	
Received counseling on contraceptive methods after HIV diagnosis	Yes	366	418	5.25(2.56,10.79)*	2.67(0.85,8.33)	0.092
	No	9	54	1.00	1.00	
Had open discussion with husband/partner about contraceptive methods and use	Yes	256	66	6.65(4.05,10.93)*	6.34(3.42,11.76)**	<0.001
	No	35	60	1.00	1.00	

*COR-Crude Odds Ratio, AOR-Adjusted Odds Ratio,*

*significant at P = 0.05,

**significant at P<0.001.

In adjusted analyses, there were no significant differences in contraceptive use by HIV clinical characteristics. Residency, education, number of living children and open discussion with sexual partner/husband had significant association with contraceptive use among HIV-positive women.

Urban residents were more likely than their rural counterparts to report contraceptive use with statistically significant difference (AOR = 2.55; 95% CI: 1.27, 5.02). Primary education (AOR = 2.27; 95% CI: 1.12, 2.86), having one or more living children, and having open discussion with husband/sexual partner about contraceptive methods (AOR = 6.34; 95% CI: 3.42, 11.76) and use (AOR = 6.34; 95% CI: 3.42, 11.76) remained significantly associated with contraceptive use (**Table 3**).

## Discussion

In this study, 44.3% of HIV-positive women were using contraceptive methods at time of survey. This finding is comparable with other studies, 48% [20] and 43% [16]. In the same region (Tigray), 46.3% of contraceptive use was reported by Berhane Y. et al, in 2013 [35]. This is due to the fact that the level of sexual activity and knowledge on contraceptive methods in these study areas are similar [16], [20]. However the proportion of HIV positive women who were using contraceptive methods in Uganda, 66.4% [15] and 80% [19] and in South Africa, 89.8% [11] were very high compared to ours. This could be due to high quality and strong integration of SRH services with ART/HIV services in these countries [11], [15], [19]. Low level of contraceptive utilization by HIV positive women will lead to high rate of unintended pregnancy, vertical and sexual transmission of the virus [9]. Attitude of HIV positive women towards contraceptive utilization as a result of misconception, culture and religious barriers can lead to low prevalence of contraceptive use [3]. Similarly, structural barriers, like low availability of methods, can influence utilization of contraceptive methods in this segment of the population [18].

The proportions of HIV positive women who were using contraceptive in Uganda, 34% [22], Swaziland, 17.9% [10], and Uganda, 25% [18], found to be lower than our study participants. Similarly, other study in Uganda reported that contraceptive utilization among HIV positive women is very low [17]. The immediate fertility desire of women in these studies was very high as it may lower contraceptive utilization [10], [17], [18], [22].

In our study, injectable was the most commonly used type of contraceptive method which accounts 70.7% of users. This is also similar with a study conducted in South Africa, 70.2% [11]. Women were interested in using injectable because it can be used without their partners' awareness and injectable has less tension than pills like swallowing and remembering of timing of pills swallowing [21]. However, utilization of injectable method reported very low in Uganda, 4.1% [17] and Northern Malawi, 19% [20]. This could be the cost of injectable is high than other methods in these countries [21] and in Ethiopia, family planning services are free of charge. A study from Tigray state has demonstrated that lower proportion (22.7%) of HIV positive women was using injectable [35]. The study setting from which the sample was taken could explain this difference. Ours was conducted in health centers unlike zonal hospital of this study.

In the current study, male condom was used by 47.6% of HIV positive women. This is slightly comparable with two studies in Uganda, 54.9% [17] and 39% [19]. However, this was higher than studies in different parts of the world. Studies in Northern Malawi, Uganda and France, indicated that 19% [20], 11% [18] and 31 [16] of HIV positive women were using condom, respectively. On the other hand, studies in Ethiopia (70%) and Uganda (90%) reported that higher proportions of women were using condom [15], [35]. In another qualitative study, most women preferred condom as method of pregnancy prevention since they concerned about taking additional pills in addition to ART [21]. In our study dual method utilization was 14.1% which is comparable with other study conducted among HIV clinic clients in Uganda, 11% [19]. Higher proportions were reported by studies in Tigray state (Ethiopia) (59.9%) [35] and Uganda, 4% [15].

Having open discussion with husband/sexual partner about contraceptive methods remained positively associated with contraceptive use. Similar findings were reported by different researches. HIV disclosure for sexual partner was also associated with increased fertility desire of women [36], [37]. Open discussion about contraceptive methods with health workers and spouse were found to be positively associated with current contraceptive utilization [18]. Similarly, a study in HIV clinics indicated that women who didn't disclose their HIV status to sexual partners and women who didn't discuss on fertility issues were less likely to use contraception [19].

Though there are wide ranges of factors which affect utilization of contraceptive methods among HIV positive women, only individual level factors were addressed in this study. Hence, considering factors from the service providers' side [38] and structural barriers [18] would have been important. In addition, male clients were not included in the study, which might be important to consider.

In conclusion, less than half proportion of HIV positive women were utilizing contraception at time of survey. Educational status, number of living children and discussion with husband/sexual partner on contraceptive methods and use, are identified as independent predictors of contraceptive use among HIV positive women. Through prevention of unintended pregnancy, integrated services are likely to benefit maternal and child health, prevent vertical transmission, and decrease incidence of conception-related sexual transmission to discordant sexual partners [39].
